# Quantification of race/ethnicity representation in Alzheimer’s disease neuroimaging research in the USA: a systematic review

**DOI:** 10.1038/s43856-023-00333-6

**Published:** 2023-07-25

**Authors:** Aaron C. Lim, Lisa L. Barnes, Gali H. Weissberger, Melissa Lamar, Annie L. Nguyen, Laura Fenton, Jennifer Herrera, S. Duke Han

**Affiliations:** 1 Department of Family Medicine, Keck School of Medicine of USC, Alhambra, CA USA; 2 Rush Alzheimer’s Disease Center, Rush University Medical Center, Chicago, IL USA; 3 The Interdisciplinary Department of Social Sciences, Bar-Ilan University, Raman Gat, Israel; 4 Department of Psychology, USC Dornsife College of Letters, Arts, and Sciences, Los Angeles, CA USA; 5 USC School of Gerontology, Los Angeles, CA USA; 6 Department of Neurology, Keck School of Medicine of USC, Los Angeles, CA USA

**Keywords:** Alzheimer's disease, Diagnostic markers

## Abstract

**Background:**

Racial and ethnic minoritized groups are disproportionately at risk for Alzheimer’s Disease (AD), but are not sufficiently recruited in AD neuroimaging research in the United States. This is important as sample composition impacts generalizability of findings, biomarker cutoffs, and treatment effects. No studies have quantified the breadth of race/ethnicity representation in the AD literature.

**Methods:**

This review identified median race/ethnicity composition of AD neuroimaging US-based research samples available as free full-text articles on PubMed. Two types of published studies were analyzed: studies that directly report race/ethnicity data (i.e., direct studies), and studies that do not report race/ethnicity but used data from a cohort study/database that does report this information (i.e., indirect studies).

**Results:**

Direct studies (*n* = 719) have median representation of 88.9% white or 87.4% Non-Hispanic white, 7.3% Black/African American, and 3.4% Hispanic/Latino ethnicity, with 0% Asian American, Native Hawaiian/Pacific Islander, and American Indian/Alaska Native, Multiracial, and Other Race participants. Cohort studies/databases (*n* = 44) from which indirect studies (*n* = 1745) derived are more diverse, with median representation of 84.2% white, 83.7% Non-Hispanic white, 11.6% Black/African American, 4.7% Hispanic/Latino, and 1.75% Asian American participants. Notably, 94% of indirect studies derive from just 10 cohort studies/databases. Comparisons of two time periods using a median split for publication year, 1994–2017 and 2018–2022, indicate that sample diversity has improved recently, particularly for Black/African American participants (3.39% from 1994–2017 and 8.29% from 2018-2022).

**Conclusions:**

There is still underrepresentation of all minoritized groups relative to Census data, especially for Hispanic/Latino and Asian American individuals. The AD neuroimaging literature will benefit from increased representative recruitment of ethnic/racial minorities. More transparent reporting of race/ethnicity data is needed.

## Plain language summary

Members of some racial and ethnic minority groups in the USA are more likely to develop Alzheimer’s Disease than white people. However, they are often not included in research studies of Alzheimer’s Disease. We looked at the race/ethnicity composition of people evaluated in papers published describing Alzheimer’s Disease research studies based in the USA that used images of the brain. We found that all racial/ethnic minority groups were underrepresented in Alzheimer’s Disease research studies, especially Hispanic/Latino and Asian American individuals. It is important that studies include representatives of all populations both for the health of those populations and improved understanding of Alzheimer’s Disease in all people. Such studies should also improve efforts to understand and address racial/ethnic disparities in Alzheimer’s Disease diagnosis and treatment.

## Abstract (web summary)

Lim et al. provide a systematic review of the race/ethnicity representation in USA based neuroimaging studies of those with Alzheimer’s Disease. Whilst sample diversity has increased in recent years, minoritized groups in the USA remain underrepresented and more transparent reporting of race/ethnicity data is needed.

## Introduction

Note on terminology: Throughout this paper, we apply the race and ethnicity categories described in the 1997 document “Office of Management and Budget Revisions to the Standards for the Classification of Federal Data on Race and Ethnicity”^1^. This includes Hispanic/Latino ethnicity, and race categories of white, Black/African American, Asian American, Native Hawaiian/Pacific Islander, American Indian/Alaska Native, Multiracial, and Other Race.

Alzheimer’s Disease (AD) is a neurodegenerative disorder with insidious onset and progressive impairment of behavioral and cognitive functions. AD can be biologically classified by three neuropathological markers. These include: (1) Aβ plaques as indicated by cortical amyloid PET ligand binding or low CSF AB_42_; (2) fibrillar tau as indicated by elevated CSF phosphorylated tau or cortical tau PET ligand binding; and (3) neurodegeneration or neuronal injury assessed as CSF T-tau, FDG PET hypometabolism, or MRI atrophy^2,3^. Symptoms of AD vary and are associated with the stage of the disease. The advanced stage of the Alzheimer’s clinical syndrome known as Alzheimer’s Dementia is characterized by impairment of memory, comprehension, language, attention, and executive function^2,4^. The estimated prevalence of AD was 1.6% of the US population in 2014, and is expected to double to 3.3% by 2060, when 13.9 million Americans are projected to have the disease^5^.

There are significant differences in AD incidence across racial/ethnic groups in the United States. Compared to Non-Hispanic white individuals, Black/African American individuals are twice as likely and Hispanic/Latino individuals are 1.5 times as likely to develop AD^6^. Among people 65 and older in the US, Black/African American individuals have the highest AD prevalence of all racial groups (13.8%), followed by Hispanic/Latino individuals (12.2%), non-Hispanic white individuals (10.3%), American Indian and Alaska Native individuals (9.1%), and Asian American and Pacific Islander individuals (8.4%)^5^. These racial and ethnic differences have multiple potential causes, including socioeconomic and educational disparities and associated stressors, healthcare access, medical comorbidities—particularly cardiovascular risk and disease, genetic risk variants, environmental exposures, and myriad other factors^7–15^.

The United States is also experiencing a demographic shift, with minority populations, classified by the US Census Bureau as racial and ethnic groups other than non-Hispanic white, projected to outpace the growth of non-Hispanic white individuals in the coming decades. An estimated 45% of the US population ≥65 years old will be from a minority group by 2060^5^. The total population of non-Hispanic whites is estimated to increase by 75%, African Americans by 172%, Asian Americans and Pacific Islanders by 270%, American Indian and Alaska Natives by 274%, and Hispanic/Latinos by 391%^16^.

Despite the differential AD risk and changing demographics, studies have consistently indicated that ethnic and racial minoritized groups are underrepresented in AD research^17–19^. Reviews have suggested that possible contributing factors mostly focus on recruitment challenges and include minoritized populations’ distrust of scientific studies based on egregious historical experimentation on African American and Native American individuals^10,20^, lack of appropriate trust building and community networking, language and literacy barriers, and insufficient resources and training to develop culturally informed recruitment and retention strategies^21^. These barriers to participation frequently overlap and vary across geographic regions and cultures^18^, and recruitment challenges are identified as the primary barrier negatively impacting AD clinical research progress^22^.

The implications of under-recruiting minoritized groups are significant. Existing studies have repeatedly reported on the lack of racial/ethnic diversity in individual clinical trials and AD research assessment databases^19,23,24^, which can impact the generalizability of findings on AD processes, biomarker cutoffs for diagnosis and normative comparisons, and effectiveness of AD treatments. A growing number of neuroimaging and biomarker studies are addressing factors like race and ethnicity^25,26^, though not all studies have identified ethnic/racial differences in the relationships between brain cognition and function^27,28^. For example, community-dwelling Mexican American individuals experience neurodegeneration at significantly younger ages than Non-Hispanic white individuals, and neurodegeneration is correlated with different health factors in these two populations^29^. Some, but not all, studies report that Black/African American individuals have lower cerebrospinal fluid levels of tau-related biomarkers than Non-Hispanic white individuals, despite similar CSF levels of AB42, NfL, and hippocampal and white matter hyperintensity volume^30–32^, and AD correlates with increased CSF IL-9 in Black/African American individuals but not white individuals^33^. Black/African American patients with elevated beta-amyloid demonstrate smaller hippocampal volume and decreased cortical thickness than comparative Non-Hispanic white patients^34,35^. These two groups also exhibit opposite patterns of Default Mode Network resting-state connectivity in AD, potentially demonstrating race-specific AD trajectories that may contribute to differential rates of cognitive decline^36^. The few studies on American Indian individuals do not show evidence of neurodegenerative risk from Apolipoprotein (APOE) E4 on intracranial volume or cognitive testing^37^ and correlations between cognition and hippocampal volume appear to be similar to Non-Hispanic white individuals^38^. The Honolulu-Asia Aging Study has found that Hawaiian Japanese American men have lower neuropathological density relative to Non-Hispanic white individuals^39,40^. Notably, for race- and ethnicity-based comparisons, these differences remain significant after accounting for other demographic variables and comorbid medical conditions.

So while acknowledgement of racial and ethnic disparities in AD clinical outcomes and neuroimaging research has increased in recent years, with corresponding calls for better representation of ethnic/racial minoritized groups in AD research to ameliorate these disparities^17,21,41^, no studies have quantified the breadth of race/ethnicity representation in the neuroimaging literature, an important step in understanding the extent of minoritized group underrepresentation in a critical area of research in the field. This descriptive review addresses this gap by identifying mean and median race and ethnicity composition of AD-related neuroimaging research samples in published works based in the United States. We accessed this information from relevant publications indexed on the PubMed database. We also examined trends over time by investigating median representation over two time periods (1994–2017 and 2018–2022), delineated by median publication year for all included studies. Overall, we identified that all racial/ethnic minority groups were underrepresented in Alzheimer’s Disease research studies, especially Hispanic/Latino and Asian American individuals, and that sample diversity has been improving over time.

## Methods

### Search strategy

This review was conducted in accordance with the Preferred Reporting Item for Systematic Review and Meta-Analyses (PRISMA) guidelines^42^ and registered on the International Prospective Register of Systematic Reviews (PROSPERO) (ID CRD42022303573). We conducted an initial literature search in January 2022 using a PubMed database from a well-resourced university account, and articles were reviewed from January through September 2022. The primary inclusion search terms included “Alzheimers” and keyword variants related to neuroimaging, including “imaging” as well as specific neuroimaging techniques (e.g., “magnetic resonance imaging” or “positron emission tomography”) in the title or abstract. We were interested in publications with neuroimaging outcomes with research samples based in the United States, and therefore excluded studies with certain key terms in the title or abstract. First, search results were refined using PubMed’s filters, and were restricted to English journal articles, Human species, non-review- or meta-analysis-type articles, and available free full-text articles. We screened out non-quantitative studies using such terms as “qualitative”, “case study”, “commentary” and “study protocol”. As many animal studies were not filtered out by the Human Species filter, we excluded studies with key words such as “animals”, “rodent”, and “cell model”. Finally, we excluded studies primarily reporting histological or post-mortem data using such key words as “postmortem” “autopsy” and “histological”, as recruitment procedures and strategies for post-mortem and in-vivo neuroimaging studies can widely differ. We did, however, include studies for which living participants were recruited and imaged, and who subsequently agreed to a body donation for post-mortem research. The full search term and filters applied are available in Supplementary Table 1.

**Fig. 1:**
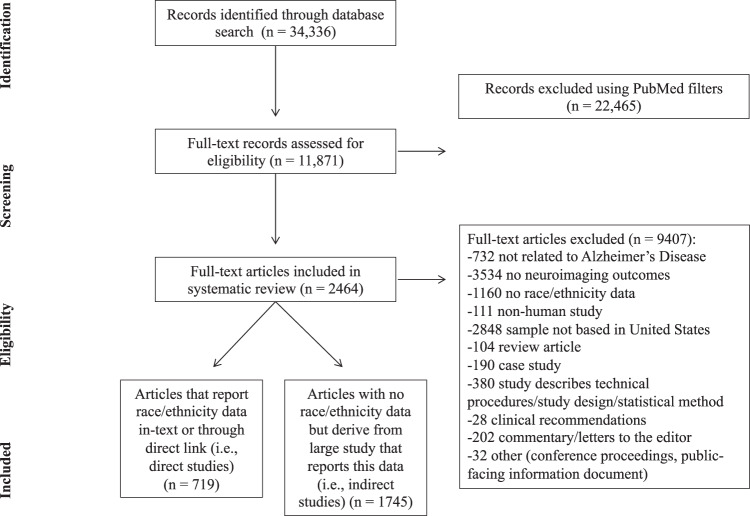
Article selection flowchart. Of 34,336 articles retrieved, 2464 articles were included in this review, divided into direct and indirect studies.

The remaining articles in the literature search were reviewed in full, as it was not always clear from abstract review whether a study recruited a sample based in the US, reported neuroimaging outcomes, or described a breakdown of race/ethnicity. Full-text articles were excluded at this stage if they did not meet the inclusion criteria, and reasons for exclusion are reported in the next section and in Fig. 1.

### Eligibility criteria

Eligible articles for this review examined associations between AD processes and neuroimaging outcomes (e.g., from MRI, PET, CT) using quantitative methods/analysis, by means of experimental or observational study, included a sample based in the US, and reported race/ethnicity sample characteristics. To ensure that race/ethnicity comparisons were consistent across studies using US census categories, only participant samples that were based in the US were included. Multisite international studies with at least one US site that reported race/ethnicity categories consistent with the US census were also eligible for inclusion; only the US site reporting race/ethnicity data in such cases was specifically included in this review. AD was conceptualized broadly to expand the breadth of this review. For instance, reports examining health conditions as risk factors for AD, such as cardiovascular disease, obesity, depression, and Down syndrome were included so long as the connection to AD was explicitly tested or stated. Studies examining cognitively unimpaired individuals/healthy aging, sex differences and/or perimenopausal changes, both amnestic and non-amnestic MCI, or non-epidemiological risk factors (e.g., genetic polymorphisms) were also included so long as the connection to AD was explicitly tested or stated. Various Stage II and III clinical trials for pharmacological treatments and PET radiotracers for AD were eligible for inclusion as well. Some studies examined associations across multiple large datasets (e.g., Alzheimer’s Disease Neuroimaging Initiative (ADNI) and Alzheimer’s Disease Cooperative Study (ADCS)) and presented race/ethnicity breakdowns for these databases separately. In these cases, each database was included as a separate study/row (e.g. one study that reports ADNI and ADCS data separately is counted as two studies, one for each dataset). Notably, studies comprised entirely of individuals with non-AD dementias (e.g., Lewy-Body/Parkinson’s Disease, frontotemporal dementia, vascular dementia) were excluded; studies examining multiple types of dementia were included if individuals along the AD spectrum (e.g., amnestic MCI) were also part of the study.

As data was formally requested from ADNI and in accordance with ADNI publishing policy: data used in the preparation of this article were obtained from the Alzheimer’s Disease Neuroimaging Initiative (ADNI) database (adni.loni.usc.edu). The ADNI was launched in 2003 as a public-private partnership, led by Principal Investigator Michael W. Weiner, MD. For up-to-date information, see www.adni-info.org.

Using these eligibility criteria, a total of 11,871 full-text articles were reviewed. Articles excluded during this stage of review met at least one of the following criteria: Not related to AD (e.g., all participants had Parkinson’s Disease, study focused on another health condition); no neuroimaging outcomes (e.g., blood biomarkers or cognition outcomes only, histological studies); non-human study; non US-based study sample; review article/meta-analysis; case study; clinical recommendations; commentary or letters to the editor; or other miscellaneous articles (e.g., summary of conference proceedings, description of technical procedure/statistical technique, consumer-facing informational pamphlet).

### Eligible studies

Studies that met all inclusion criteria fell into one of two groups. A portion of studies, hereafter referred to as “direct studies”, reported the race/ethnicity breakdown of their sample either within the text or to a direct link that had this information (e.g., to a clinicaltrials.gov link that reported the race/ethnicity composition of the sample). A second group of studies, hereafter referred to as “indirect studies”, did not report race/ethnicity information but instead described a larger study or database from which the study sample derived. For these larger studies/databases, we obtained demographic characteristics of the larger cited study/database using either formal data requests (e.g., Alzheimer’s Disease Neuroimaging Initiative), or recently published manuscripts using these datasets that reported race/ethnicity information. Use of such data for this review was consistent with consents and data access approvals from formal data requests.

### Data extraction

The following study characteristics were extracted from eligible full-text articles: year of manuscript publication, total sample size, and direct/indirect study classification (i.e., whether race/ethnicity data was directly from the study/a proximal link or if it was generalized from a larger database). Sample sizes and percentages of total samples were also recorded for the following race/ethnicity categories based on the 1997 US Census: Hispanic/Latino ethnicity; white; Black/African American; American Indian/Alaska Native; Asian American; Native Hawaiian/Other Pacific Islander; Multiracial; Some Other Race; and Unknown Race^43^. Unknown race was included because the majority of studies did not elaborate upon race/ethnicity breakdowns for non-white categories.

For studies that only reported *N*s of race/ethnicity categories, percentages for each category were calculated, while approximate *N*s were calculated for studies that only reported percentages. Many studies reported *N*s/% only for white participants. In these instances, as we could not determine the breakdown of other race/ethnic groups, the remaining non-white *N*/% was included as “Unknown” (e.g., 80% white, 20% Unknown). Participants who were classified as “Caucasian” in some studies were included in the “white” race category, consistent with historical usage of the term in medical literature^44^. Individuals who were classified as “European” or of European ancestry were coded in the Unknown race category, as race and ethnicity classifications do not consistently map onto genetic ancestry and particularly for European ancestry^45^. Studies differed in their approach to reporting Hispanic/Latino Ns/%. Multiple studies reported race and ethnicity separately, consistent with Census reporting of Hispanic/Latino ethnicity. Some studies reported Hispanic/Latino as a race category that summed with the other race categories to 100% (e.g., 34% white, 36% Black, 30% Hispanic/Latino, 0% Other). In the above example, it was therefore presumed that the white and Black individuals were non-Hispanic based on the sum total of 100%. Many studies reported race but no ethnicity data, while some studies with 100% white participants specifically stated that their white participants were non-Hispanic white; in the latter example, we therefore tabulated the study as 0% Hispanic/Latino. In ~2% of studies that reported race data, the reported sample size differed from the sum of *N*s’ in race categories, even after accounting for cases in which Hispanic/Latino ethnicity was counted as race in some studies (e.g., reported total *N* = 1000, sum of race/ethnicity = 940). As total *N* was used to calculate percentages, a primary outcome in this review, we utilized the summed total race *N* (e.g., we used *N* = 940 rather than *N* = 1000 in the above hypothetical example) rather than the reported total *N* in these instances to more accurately reflect race/ethnicity percentages of individual studies.

### Analyses

This review focuses on understanding race/ethnicity composition of research samples in US-based AD neuroimaging research. For direct studies, we therefore utilized scatterplots and histograms to understand: (1) distribution of race/ethnicity across relevant studies using median and/or mean; (2) racial/ethnic composition (i.e., % race/ethnicity) of study samples as a function of time (i.e., publication year). For this second aim, we utilized a median split on publication year for all studies. This yielded two time periods, 1994–2017 (*n* = 361 studies) and 2018–2022 (*n* = 358 studies) to compare race/ethnicity data for direct studies.

We separately examined the histograms and obtained mean/medians for larger databases cited in indirect studies that did not directly report race/ethnicity breakdowns of their samples. We also tabulated the number of times such larger databases were cited in indirect studies.

A risk of bias analysis within and across studies was considered, but race/ethnicity composition is not a modifiable outcome variable and it cannot be determined within the scope of this review whether studies or authors self-selected into reporting race/ethnicity compositions of study samples. The vast majority of studies also reported demographics for participants that met study-specific inclusion criteria rather than demographics of respective screening samples, and we therefore could not determine whether study-specific screening procedures influenced final race/ethnicity compositions.

### Reporting summary

Further information on research design is available in the Nature Portfolio Reporting Summary linked to this article.

## Results

### Literature search

A total of 2,464 articles were included after the full-text screening process. Of these, there were 719 direct studies reporting race/ethnicity data directly in-text or through a direct link, and 1745 indirect studies that did not report race/ethnicity data but derived from a larger database/cohort study that did report this data. Indirect studies were drawn from a total of 44 databases/studies.

### Articles directly reporting race/ethnicity data

Among direct studies, there was variability in the type of race/ethnicity data reported (see Table 1). The vast majority of articles (*n* = 715/719) reported white race data, with fewer numbers of studies reporting non-white races/ethnicities (*n*s = 281 of 719 studies for Hispanic/Latinos to 467 of 719 studies for Black/African Americans). About 36% of studies reported Non-Hispanic white data. Similar patterns were observed for indirect studies derived from larger databases. These larger studies/databases reported white race data, but fewer studies/databases reported other racial/ethnic compositions (*n*s = 21 of 44 databases for Native/Hawaiian/Other Pacific Islanders to 30 of 44 databases for Black/African Americans), and less than half reported Non-Hispanic white data (*n* = 18/44). The median sample size was 231, with a range of 11 to 19,309 participants.

**Table 1:** Race/Ethnicity of Alzheimer’s Disease neuroimaging studies directly (*n* = 719) and indirectly (*n* = 44 cohort studies) reporting this data.

Race/Ethnicity	N(%)	Median %	Mean %
**Direct Studies (*n*** = **719)**			
Hispanic/Latino	281(39%)	3.39	14.87
White	715(99%)	88.85	79.07
Non-Hispanic White	257(36%)	87.40	71.44
Black/African American	467(65%)	7.32	14.68
American Indian/Alaska Native	375(52%)	.00	.70
Asian American	397(55%)	.00	2.87
Native Hawaiian/Other Pacific Islander	360(50%)	.00	.04
Multiracial	360(50%)	.00	.25
Some Other Race	357(50%)	.00	.14
Unknown	717(99%)	.66	4.71
**Indirect Studies (*n*** = **1745)**			
Hispanic/Latino	25(57%)	4.67	10.72
White	44(100%)	84.18	78.92
Non-Hispanic White	17(39%)	83.28	73.44
Black/African American	30(68%)	11.59	14.29
American Indian/Alaska Native	22(50%)	.00	.26
Asian American	29(66%)	1.75	2.98
Native Hawaiian/Other Pacific Islander	21(48%)	.00	.10
Multiracial	22(50%)	.00	.66
Some Other Race	21(48%)	.00	.11
Unknown	44(100%)	.77	4.04

**Note**. Indirect Studies (*n* = 1745) derive data from cohort studies/databases (*n* = 44) that are reported in this table. Race/Ethnicity categories derived from the 1997 United States Census. *N* represents the number of studies reporting respective race/ethnicity information. Median and Mean % represent the median and mean study’s percentage composition for each race/ethnicity category.

**Fig. 2:**
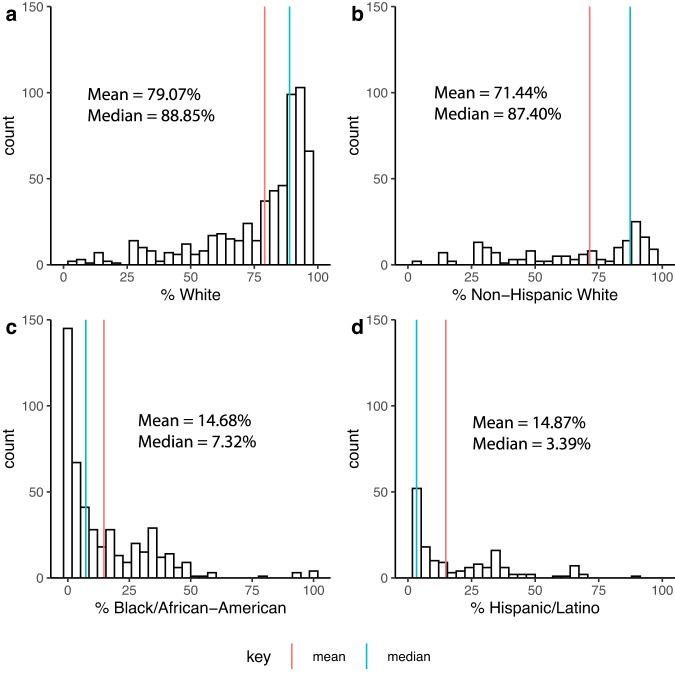
Histogram of the racial/ethnic composition of Alzheimer’s Disease neuroimaging studies directly reporting race/ethnicity data. Mean and median % representation of (**a**) white; (**b**) Non-Hispanic white; (**c**) Black/African American; and (**d**) Hispanic/Latino participants for studies directly reporting race/ethnicity data (*n* = 719). Count represents the number of studies reporting a given %.

The distribution of race/ethnicity data reported across studies was skewed for all categories (see Fig. 2). Both mean and median % race/ethnicity for all studies was calculated, but median % is reported as the optimal measure of central tendency in light of this skew. Among direct studies, the median study was 88.9% white (87.4% Non-Hispanic white), 7.3% Black/African American, and 3.4% Hispanic/Latino ethnicity (Fig. 2). Asian American, American Indian/Alaska Native, Native Hawaiian/Other Pacific Islander, Multiracial, and Some Other Race are not depicted in these figures, but had median representation of 0.00%. Median Unknown race was 0.7%, and may be slightly elevated due to the number of studies that solely reported % white race. Notably, 54% of direct studies (*n* = 390/719) investigated race/ethnicity as a primary aim, and/or included statistical analyses of race/ethnicity (e.g., between diagnostic groups, as a covariate, or in stratified comparisons).

### Race/ethnicity over time

**Fig. 3:**
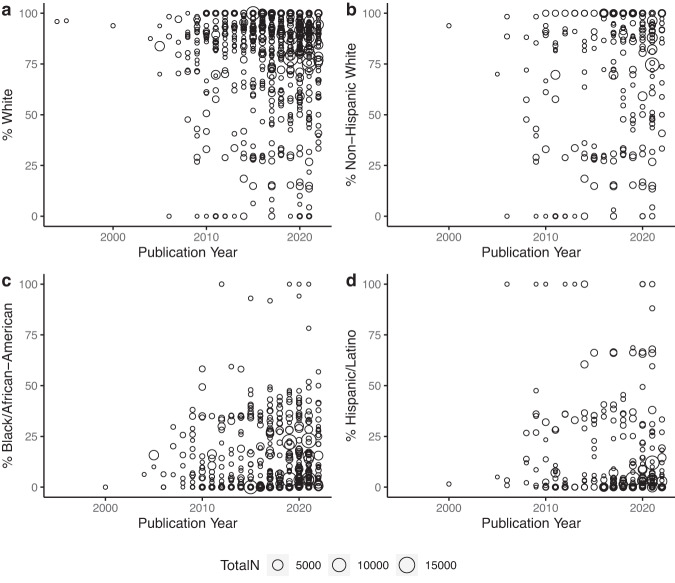
Study race/ethnicity composition by publication year for Alzheimer’s disease neuroimaging studies directly reporting race/ethnicity data. Time by % representation of (**a**) white; (**b**) Non-Hispanic white; (**c**) Black/African American; and (**d**) Hispanic/Latino participants for studies directly reporting race/ethnicity data (*n* = 719). Each study represents a dot in this scatterplot. % Race refers to % of participants in each study. Total N (dot size) refers to the sample size of each study.

A visualization of race/ethnicity data over time is shown in Fig. 3, with a summarized depiction in Supplementary Fig. 1. While formal analyses were not conducted due to likely violations of multiple tests’ assumptions (i.e. non-independent observations and lack of information about study-specific nested data), at minimum there has been an increase in the number of studies in which Non-Hispanic white participants comprised <50% of the sample; the first such AD neuroimaging study in this review was published in 2006, and 13 such studies were published in 2021. Approximately half of the 90 articles with <50% white composition from 1994–2022 were published since 2017. Median race and ethnicity % using a median split for publication year are also shown in Table 2. Comparisons of the two periods from 1994–2017 and 2018–2022 indicated similar median % race and ethnicity percentages and interquartile ranges across the two time periods. The only notable change may be for median % Black/African American, which was 3.39% from 1994–2017 and 8.29% from 2018–2022.

**Table 2:** Median-split publication year by race/ethnicity for direct studies (*n* = 719).

Race/Ethnicity	Time 1: 1994–2017			Time 2: 2018–2022		
	Median %	*N*	Interquartile Range	Median %	*N*	Interquartile Range
White	89.45%	360	71.84–96.06%	88.53%	355	71.54–94.18%
Non-Hispanic White	86.40%	128	31.40–100%	87.50%	129	50.05–99.15%
Hispanic/Latino	3.39%	133	0.00–28.34%	3.37%	148	0.00–18.39%
Black/African American	5.56%	229	0.00–25.83%	8.29%	238	2.08–27.73%
Asian American	0.00%	187	0.00–0.83%	0.00%	210	0.00–1.69%
American Indian/Alaska Native	0.00%	180	0.00%	0.00%	195	0.00%
Native Hawaiian/Other Pacific Islander	0.00%	175	0.00%	0.00%	185	0.00%
Multiracial	0.00%	176	0.00%	0.00%	184	0.00%
Some Other Race	0.00%	176	0.00%	0.00%	181	0.00%
Unknown	0.58%	360	0.00–6.54%	0.67%	357	0.00–6.58%

**Note**. Median-split on publication year produces two time periods, 1994–2017 and 2018–2022. *N* represents the

number of studies reporting respective race/ethnicity information. Median % represent the median study’s percentage composition for each race/ethnicity category.

### Indirect studies derived from larger databases

**Fig. 4:**
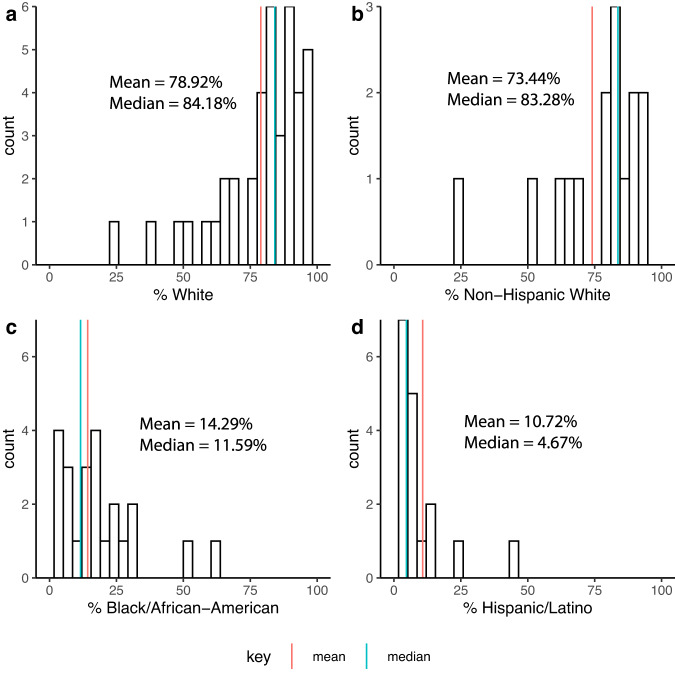
Histogram of the racial/ethnic composition of Alzheimer’s Disease neuroimaging studies indirectly reporting race/ethnicity data. Mean and median % representation of (**a**) white; (**b**) Non-Hispanic white; (**c**) Black/African American; and (**d**) Hispanic/Latino participants for cohort studies/databases (*N* = 44) from which studies indirectly report race/ethnicity data (*n* = 1745). Count represents the number of cohort studies reporting a given %.

Indirect studies (*n* = 1745) derived from 44 larger databases/cohort studies and race/ethnicity was similarly skewed as direct studies (see Fig. 4). Among these 44 databases, median representation was 84.2% white (83.7% Non-Hispanic white), 11.6% Black/African American, 4.7% Hispanic/Latino, and 1.75% Asian American (Table 1**;** Fig. 4). American Indian/Alaska Native, Native Hawaiian/Other Pacific Islander, Multiracial, and Some Other Race had median representation of 0.00%. Median Unknown Race was 0.8%. The 44 databases/studies are shown in Table 3, with ~70% of the 1745 indirect studies drawing data from the Alzheimer’s Disease Neuroimaging Initiative (ADNI), and 94% drawing from 10 databases. The median sample size of these larger databases was 1575, with a range of 57 to 45,923 participants.

**Table 3:** Number of studies that indirectly report race/ethnicity data (*n* = 1745) and cite larger databases.

Study/Database	Abbreviation	# of Studies
Alzheimer’s Disease Neuroimaging Initiative	ADNI	1219
Mayo Clinic Study of Aging	MCSA	103
Harvard Aging Brain Study	HABS	63
Open Access Series of Imaging Studies	OASIS	57
Framingham Heart Study	FHS	51
Dominantly Inherited Alzheimer Network	DIAN	46
Wisconsin Registry for Alzheimers Prevention	WRAP	43
Religious Orders Study/Memory and Aging Project	ROSMAP	24
Baltimore Longitudinal Study of Aging	BLSA	22
National Alzheimer’s Coordinating Center—Uniform Data Set	NACC UDS	14
University of Kansas Brain Aging Project	UKBAP	10
Dallas Lifespan Brain Study	DLBS	9
Human Connectome Project—Aging	HCP-A	9
BIOCARD Study	BIOCARD	7
Cardiovascular Health Study	CHS	7
A4 Study	A4	6
Atherosclerosis Risk in Communities—Neurocognitive Study	ARIC-NCS	5
Kronos Early Estrogen Prevention Study	KEEPS	5
Arizona APOE Cohort	AAC	5
Oregon Brain Aging Study	OBAS	4
Cache County Study on Memory in Aging	CCSMA	4
Central Control of Mobility in Aging	CCMA	4
Washington Heights Inwood Columbia Aging Project	WHICAP	3
The 90+ Study	–	3
Ginkgo Evaluation of Memory Study	GEM	2
Reference Ability Neural Network Study	RANN	2
EXPEDITION-3	–	1
The Aging Brain Study	ABS	1
MISSION AD	MISSION AD	1
Investigation Into Delay to Diagnosis of Alzheimer’s Disease with Exelon	InDDEx	1
Dartmouth Memory and Aging Study	DMAS	1
Action to Control Cardiovascular Risk in Diabetes—Memory in Diabetes	ACCORD MIND	1
Einstein Aging Study	EAS	1
Chicago Health and Aging Project	CHAP	1
Vietnam Era Twin Study of Aging	VETSA	1
Coronary Artery Risk Development in Young Adults	CARDIA	1
UC Davis Aging Diversity Cohort	UCD ADC	1
Knight Alzheimer Disease Research Center	Knight ADRC	1
Imaging Dementia—Evidence for Amyloid Scanning	IDEAS	1
UCSF Hillblom Aging Network	UCSF HAN	1
Monongahela-Youghiogheny Healthy Aging Team	MYHAT	1
Intelligent Systems for Assessment of Aging Changes	ISAAC	1
Sacramento Area Latino Study on Aging	SALSA	1
Genomics Superstruct Project	GSP	1

## Discussion

This descriptive review of AD neuroimaging studies examined race/ethnicity composition of US-based research samples in a PubMed search of English, free full-text articles published until September 2022. We identified 2459 articles, of which 719 were direct studies reporting race/ethnicity data directly and 1745 were indirect studies that did not report this data, but instead derived from a cohort study/database that reported this information. Median representation of race/ethnicity data from direct studies was 88.9% white and 87.4% Non-Hispanic white, 7.3% Black/African American, and 3.4% Hispanic/Latino ethnicity, with 0% median representation of other race categories. The 44 databases from which 1745 indirect studies derived race/ethnicity data tended to be slightly more diverse, with the median database comprised of 84.2% white, 83.7% Non-Hispanic white, 11.6% Black/African American, 4.7% Hispanic/Latino, and 1.75% Asian American participants. Median representation of American Indian/Alaska Native, Native Hawaiian/Other Pacific Islander, and Multiracial were 0%.

Comparisons of this data with census information on US adults age 65 and older shed light on underrepresentation issues. The most recent census report on older Americans in 2020 indicated that 76% of older adults were Non-Hispanic white, 9% Black/African American, 9% Hispanic ethnicity, 5% Asian American, 0.6% American Indian/Alaska Native, 0.1% Native Hawaiian/Pacific Islander, and 0.8% multiracial^46^. Based on current older adult data, Hispanic/Latino and Asian American individuals demonstrate the greatest disparity between census representation and median AD neuroimaging study participant representation, followed closely by Black/African Americans, who demonstrate the highest risk of AD amongst all racial and ethnic groups^5^. Further, multiracial, American Indian/Alaska Native, and Native Hawaiians/Pacific Islander individuals essentially have no reported representation in AD neuroimaging study samples. Beyond comparisons with current cross-sectional race and ethnicity census data, the overall number of older adults and proportion of older adults who are ethnic/racial minorities has been and is expected to continue increasing in the US; approximately 13% of older adults were race/ethnic minorities in 1990, 15%, 20%, and 24% in 1999, 2009, and 2019, respectively, and this share is estimated to increase to 34% by 2040^46–48^. On a basis of minority group composition, median race/ethnicity representation from studies in this review most closely approximate Census data from 1990 and 1999 for direct and indirect studies, respectively.

Racial/ethnic group representation in AD neuroimaging studies in the US is low but may be slowly improving over time. Comparing median sample compositions from 1994–2017 and 2018–2022 indicates that median % white (89.5% and 88.5%) and Non-Hispanic white (86.4% and 87.5%) remained relatively unchanged in direct studies, despite increased ethnic/racial diversity in older adult census data. The one promising representation change is that median % Black/African American participants in research samples may have improved slightly from 3.4% to 8.3% during these two time periods. Increasing representation of Black/African American individuals is particularly important, given elevated risks of AD among Black/African American populations and repeated calls for improving representation of this group in AD research^49,50^. The proportion of another minoritized group with high AD incidence, Hispanic/Latino individuals, remained static between these two time periods (3.4% and 3.4%), which further underscores severe underrepresentation of Hispanic/Latino individuals in AD neuroimaging research. Median representation of all other racial groups (Asian Americans, American Indian/Alaska Natives, Native Hawaiian/Other Pacific Islanders, and multiracial) has been consistently low, with median 0.00% representation across the two time periods. Overall, these trends appear to demonstrate that published studies retain similar levels of majority white samples over time, though the proportion of studies with more African-Americans may be increasing in recent years. Further, there is a small but growing number of studies that focus on minority recruitment and are composed of <50% white participants, with one such publication in 2006 and 13 in 2021.

Overall trends in diversifying US research populations may also be reflected in the relatively greater diversity in indirect study databases relative to direct studies. These 44 databases appeared to have a lower median proportion of white participants relative to the 719 direct studies (84.2% white vs 88.9%; 83.7% Non-Hispanic white vs 87.4% Non-Hispanic white), and greater median numbers of Black (11.6% Black vs 7.3% Black), Hispanic/Latino (4.7% vs 3.4%) and Asian American (1.75% vs 0.00%) participants, though American Indian/Alaska Natives and Native Hawaiians/Other Pacific Islanders were again shown to have essentially zero representation. This greater relative diversity may be attributable to cohort studies’ active efforts to improve participant diversity in recent years, as race/ethnicity composition data was acquired for databases’ current participant numbers using formal data requests or recently published articles that contain this information. Large cohort studies of minority groups are increasingly recruiting participants, such as the Minority Aging Research Study^51^ and Latino CORE study at Rush University^52^, UC Davis Diversity Cohort^53^, the Health & Aging Brain among Latino Elders Study^54^, University of North Texas Health Science Center’s Black Alzheimer’s Brain Study (https://blackalzbrainstudy.com/), and the Study of Latinos—Investigation of Neurocognitive Aging (SOL-INCA)^55^. New initiatives in Alzheimer’s research participant registries target specific groups, such as the Collaborative Approach for Asian Americans and Pacific Islanders Research and Education Registry (https://www.alzheimers.gov/clinical-trials/care-registry-asian-americans-and-pacific-islanders-0). The African American Outreach Satellite has been successful in doubling African American enrollment as a part of Knight Alzheimer’s Disease Center at Washington University St. Louis^56^. These efforts include aspects of relationship-building within racial/ethnic communities and cultural institutions therein, multilingual materials and resources, focused advertisement and recruitment campaigns, hiring of diverse research staff, and sharing of research findings through public talks. Expanding such research efforts to strengthen and maintain relationships with such communities may be critical to the race/ethnicity representation improvements seen over time.

Increasing diversity recruitment efforts in ADNI may also reflect recent enrollment trends in large databases that will have impacts on AD research in the US. ADNI-1 was completed in 2010 and comprised of 90.4% Non-Hispanic white participants, while ADNI is 84.3% Non-Hispanic white across all phases from currently available baseline data (ADNI, 2022). ADNI4, the next phase of ADNI, aims to further improve on race/ethnicity composition by recruiting 50–60% of its new participants from underrepresented populations, including racial/ethnic minorities and those from lower socioeconomic backgrounds^57^. Further, the most recent ADNI publication indicates a total ADNI sample of 79.3% white, 11.5% Black, 5.6% Latinx, 2.7% Asian, 0.8% Native American, and 0.5% Other Race individuals^57^. Of note, approximately 70% of the 1745 indirect studies utilized ADNI data. If articles in this review reasonably reflect the US AD literature, large databases like ADNI have a responsibility to diversify samples not only to address historical underrepresentation, but also expand inquiry into mechanisms that increase AD vulnerability in these groups in light of how frequently ADNI data is published. ADNI has been critical to elucidating AD processes^58^, refining statistical techniques and predictive models^59^, and identifying targets for longitudinal treatment effects and biomarker thresholds^60–62^. ADNI data has been combined with other large population studies to examine genetic AD risk factors among Non-Hispanic white populations^63^. Expanding the number of racial/ethnic minorities in ADNI will provide rich datasets that allow for similar such investigations and cross-study comparisons across race/ethnicity groups in the US.

Improved recruitment of racial and ethnic minoritized groups is needed beyond large cohort databases in the US. Such large-scale volunteer databases are frequently used for complex data analyses that elicit conclusions assumed to be broadly applicable. In this review, 94% of indirect studies drew data from 10 such databases. The AD research community demonstrates significant density in social network structure^64^, and increasingly focuses on big data to advance AD research goals^65^. These volunteer databases are not frequently representative of general populations, however, in that participants are more likely to be female, less likely to live in socioeconomically deprived areas, are better educated, have higher incomes, and require fewer medications^66–70^. Large scale-volunteer databases require substantial resources and increasingly dominate funding and publication proportions across fields, but may not be well-equipped to address inequalities in health and aging^71^. Some in AD research have acknowledged these shortcomings^72–74^, and many existing epidemiologic US population-based cohorts that examine AD are not racially/ethnically diverse. Nationally representative surveys have identified race and ethnicity-specific risk factors for AD, including midlife obesity for American Indian and Alaska Native individuals, Black individuals, and white individuals; low years of education for Hispanic/Latino individuals, and physical inactivity for Asian American individuals^14^. AD neuroimaging studies may similarly benefit from diverse representation to examine race/ethnic disparities in risk factors and adverse experiences like stressful life events or medical comorbidities. This is especially relevant if adversity and other sequelae contribute to AD risk and impact the likelihood of receiving specialized AD treatment^75,76^. Representativeness may also be needed to understand heterogeneity in experiences within racial/ethnic groups contributing to differential AD outcomes. Efforts to improve representativeness of samples that contribute data to large-scale efforts like the US National Alzheimer’s Coordinating Center (NACC), in addition to smaller cohort samples that focus on representativeness can reduce potential bias by recruiting ethnic/racial minoritized individuals across a spectrum socioeconomic status, education, and health conditions.

AD neuroimaging studies in the US should also more openly publish race/ethnicity composition of research samples, and indicate data sources in secondary analyses. In this review, 719 articles reported this data directly and 1745 indirectly from a larger database, while an additional 1160 articles met study criteria but neither reported data directly nor cited a specific database from which the study derived. Approximately 20% of studies that met criteria for inclusion in this review therefore directly reported this information. Further, 64% of studies that directly reported race/ethnicity data did not report data for at least one race/ethnicity category, and 99.4% of direct studies reported data on white participants. Approximately a third of direct studies did not report where their data was sourced, and we therefore could not determine whether studies reflect potential geographic disparities in AD neuroimaging research representation. Transparency and reporting of this data using established guidelines^44^ is needed to understand the landscape of race and ethnic group representation in US AD neuroimaging research.

This descriptive review has strengths and limitations. Strengths include a review of all available articles with appropriate parameters on a well-indexed publication search, consideration of studies that directly report race/ethnicity and those that drew from larger databases that report this information, and examination of race/ethnicity composition over time. While race/ethnicity disparities in study representation have been acknowledged for decades, this is the first study to broadly quantify race/ethnicity representation in Alzheimer’s neuroimaging research in the United States. We believe the articles included in this review reasonably reflect the AD neuroimaging research literature in the United States. There are also important limitations. We specifically reviewed free full-text English language articles using a single database, PubMed, with a focus on US-based samples. Such a constrained search may not have captured all articles that met this review’s inclusion criteria, and limits the generalizability of these results. We did not search across multiple databases or scour references for additional relevant articles; it became clear after PROSPERO registration that PubMed was a more appropriate database for this review than PsycInfo given the biomedical nature of Alzheimer’s Disease. Further, the conclusions drawn from median percentages calculated from this review assumed race/ethnicity reporting is not biased; it is unknown if articles that did not report this data were more or less racially/ethnically diverse than articles that did report this data. Similarly, the data on 44 large databases likely underreports the number of existing AD neuroimaging databases, and may not fully capture data from large databases in this research literature. Considering missing information about data sources across studies that reported race/ethnicity data, data underlying direct studies in this review are likely, at least in part, nested. Due to the non-independent observations of percent race/ethnicity per study, a formalized statistical analysis of studies over time was not feasible. An inherent assumption in this review is that studies were consistent in how they captured participants’ race/ethnicity data. The United States Census has previously changed the form of the question inquiring about Hispanic ethnicity, which contributed to shifts in individuals’ responses, most notably between the 2000 and 2010 Census^77^. This review cannot determine whether questions inquiring about race/ethnicity were similar across studies. Not all studies reported race/ethnicity data for all categories, with most studies primarily reporting data from white participants. Increased transparency for future studies is needed to ensure fair and comparable percentages for race/ethnicity. Understanding representation of some minoritized groups may be limited due to the nature of US Census race/ethnicity categories. Middle-Eastern and North African (MENA) individuals are categorized as “white” on the US Census, though there is increasing evidence that a MENA label may more accurate to their lived experiences^78^. Hispanic/Latino individuals have historically self-reported as “Some Other Race” in research and population studies to expound on racial identities that do not conceptually align with US race taxonomy categories, with 45.3 million Hispanic/Latino individuals doing so in the 2020 Census^79^. Hispanic/Latino individuals may have an undercount rate of 4.99% from the 2020 Census, which further underscores Hispanic/Latino underrepresentation in AD neuroimaging research^80^. Future similar reviews across countries may be helpful to delineate race/ethnic diversity across countries and inform international collaborative AD research efforts.

Overall, this review demonstrates that racial/ethnic minoritized groups in AD neuroimaging research in the United States have been historically underrepresented, but diversity may be slowly improving in recent years, particularly for Black/African American participants. Understanding the landscape of Alzheimer’s Disease representation in studies is necessary to contextualize progress in correcting these imbalances and historical failures.

## Supplementary information

Supplementary Information

Reporting Summary

## Data Availability

Data used in this manuscript and used to plot the figures can be accessed from: https://github.com/aaronclim/adreview^81^. (10.5281/zenodo.8096807)

## Code availability

Code for generating figures can be accessed from: https://github.com/aaronclim/adreview^81^. 10.5281/zenodo.8096807
