# Supplementary material for: Quantification of race/ethnicity representation in Alzheimer’s disease neuroimaging research in the USA: a systematic review
Source: Commun Med (Lond). 2023 Jul 25;3:101. doi: 10.1038/s43856-023-00333-6 (PMC10368705; doi:10.1038/s43856-023-00333-6)
Supplement: Supplementary file 1 — Supplementary Information [file 43856_2023_333_MOESM1_ESM.pdf]

Supplementary Information for Lim et al., Quantification of Race/Ethnicity Representation in Alzheimer's Disease Neuroimaging Research in the USA: A Systematic Review

Supplementary Table 1. Search term for pubmed database

Term
alzheimers AND (imaging OR cortical OR subcortical OR neuroimaging OR "resting state" OR "network" OR T1 OR T2 OR "gray matter" OR "white matter" OR "functional connectivity" OR "functional magnetic resonance imaging" OR fMRI OR "magnetic resonance imaging" OR MRI OR "Diffusion weighted imaging" OR DWI OR "computerized tomography" OR CT OR "Magnetic Resonance Spectroscopy" OR MRS OR "Single photon emission computed tomography" OR SPECT OR "Diffusion tensor imaging" OR DTI OR "structural imaging" OR "Positron emission tomography" OR PET OR "magnetization transfer imaging" OR MTI OR "Voxel-based morphometry" OR VBM OR "Electroencephalography" OR EEG OR "magnetoencephalography" OR MEG OR "functional near-infrared spectroscopy" OR FNIRS) NOT (Review[Publication Type]) NOT (Meta-Analysis[Publication Type]) NOT "qualitative" NOT "case study" NOT "autopsy" NOT "postmortem" NOT "animal" NOT "study protocol" NOT "study design" NOT "commentary" NOT "case report" NOT "systematic review" NOT "mouse" NOT "rat" NOT "rodent" NOT "cell model" NOT "cell models" NOT "cellular model" NOT "in silico" NOT "histology" NOT "histological"

Filters Applied
"English language"
"Human Species"
"Free Full Text"

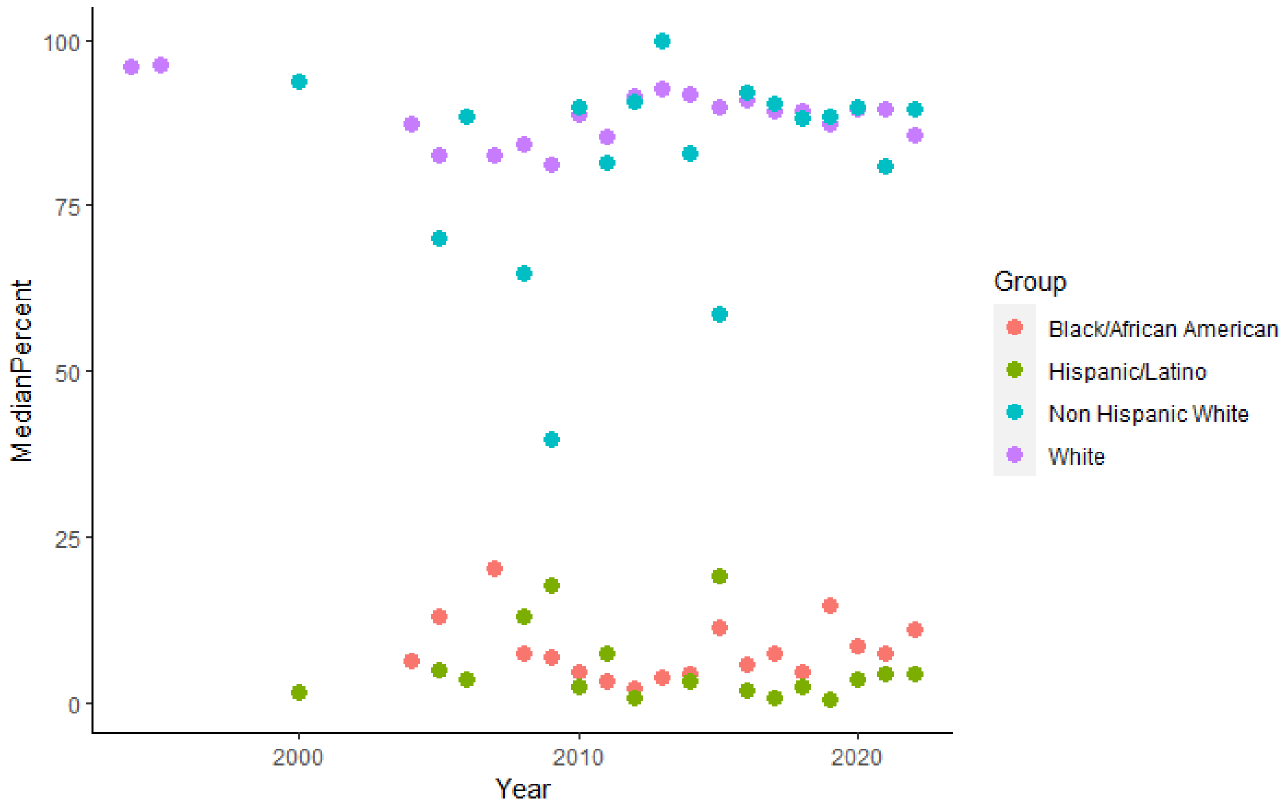

Supplementary Figure 1. Median percent race/ethnicity of direct studies per publication year. Each dot represents the median percent race/ethnicity per study for a given publication year (e.g. 2010). Data represents studies that directly reported race/ethnicity in-text or through a direct link.
